# Family life under strain: the impact of forced migration on refugee parenting in reception centers

**DOI:** 10.3389/fpsyg.2025.1554692

**Published:** 2025-05-07

**Authors:** Jennifer Drummond Johansen, Sverre Varvin, Mette Sagbakken

**Affiliations:** ^1^Department of Social Work, Child Welfare and Social Policy, Faculty of Social Sciences, Oslo Metropolitan University, Oslo, Norway; ^2^Department of Nursing and Health Promotion, Faculty of Health Sciences, Oslo Metropolitan University, Oslo, Norway

**Keywords:** forced migration, parenting, resilience, parent–child relations, parenting practices, refugees and asylum seekers

## Abstract

**Introduction:**

The global refugee crisis has reached unprecedented levels, with millions, including a significant number of children, being displaced from their homes. Refugees face mental health challenges due to pre-migration trauma, adverse flight experiences, and post-migration stressors which severely impact family dynamics and parenting practices. This study explores the effects of forced migration on refugee parents living in Norwegian reception centers, focusing on how the context of the reception centers influence parenting capacities.

**Methods:**

The research adopts ecological and sociocultural perspectives to understand resilience and parenting within the context of forced migration. Data collection includes individual interviews with 12 parents, field notes, and reflexive accounts, analyzed using Interpretative Phenomenological Analysis (IPA) to capture the participants’ lived experiences.

**Results:**

Findings indicate that the constraints imposed by the asylum system significantly hinder parents’ access to essential resources, leading to increased stress and reduced parenting capacities. Parents reported feelings of being trapped in a state of uncertainty due to a lack of stability and structure. This exacerbated their mental health issues and impaired their ability to provide a secure and nurturing environment for their children. Many parents expressed feelings of isolation and powerlessness.

**Discussion:**

The study highlights the need for host societies to address systemic barriers and provide comprehensive support to refugee families. Interventions should focus on creating a “safe haven” that facilitates psychosocial rehabilitation and supports the well-being of both parents and children. Understanding the broader socio-political context and its impact on refugee parenting practices is crucial and requires shifting from a deficit model of parenting to one that recognizes the resilience and strengths of refugee parents.

**Conclusion:**

This research offers a deeper understanding of the complex challenges faced by refugee parents in reception centers. It underscores the necessity for tailored interventions that address both individual and systemic factors to foster resilience of refugee families. Further studies are essential to explore the long-term effects of forced migration on parent–child relationships and child development, and to develop interventions that support the unique needs of refugee families in various contexts.

## Introduction

In an era where we are witnessing record levels of displacement, the UNHCR reports an alarming 120 million individuals worldwide have been forced from their homes, with nearly half being under the age of 18 (UNCHR, 2023). The detrimental impact of pre-migration traumatization, adverse flight experiences, and post-migration stressors on the mental health of refugee populations is well-established (Ermansons et al., 2023). Studies across refugee receiving countries both in Europe and globally document high levels of psychological distress and psychopathology (Leiler et al., 2019; Henkelmann et al., 2020; Solberg, 2020; Charlson et al., 2019). Political violence and war, the predominant causes of forced migration, uproot individuals, and family attachment structures and community connections (Papadopoulos, 2018; Bunn et al., 2023; De Haene et al., 2010). Forced migration has a major impact on all facets of family life, and an expanding literature documents that parents serve as potential powerful sources of both risk and resilience to the adjustment, well-being and mental health of refugee children (Betancourt and Khan, 2008; Scharpf et al., 2020; Mooren et al., 2023). Refugee children rely on caregivers to provide a sense of stability and security to cope with adverse experiences and stressors they are confronted with in the process of adjusting to an uprooted family life (Betancourt et al., 2013). However, the capacity of refugee families to create an environment that is attuned to the child’s needs is under exceptional pressure (Johansen and Varvin, 2019; De Haene et al., 2010).

Refugees encounter numerous stressors throughout their journey. The flight itself exposes refugees to traumatizing conditions (violence and other kinds of adversity), and poor living conditions with many asylum seekers being forced to make dangerous journeys and crossings by land or sea. Upon arrival, they often face language barriers, cultural differences, and feelings of isolation and insecurity. Ongoing exposure to stressors has been shown to significantly increase caregiver stress, which in turn may compromise their ability to attend to their children’s needs (Critelli, 2015; El-Khani et al., 2018). The phenomenon of intergenerational transmission of trauma highlights the indirect impact of parental hardships on children’s outcomes, elucidating the potential influence of parents’ traumatic experiences on their children’s development and well-being (van Ee et al., 2012). A systematic review and meta-analysis by Eltanamly et al. (2021) showed that more war-exposed parents showed less warmth and more harshness toward their children. This partly mediated the association between war exposure and child adjustment, such as post-traumatic stress symptoms, depression and anxiety, social problems, and externalizing behavior (Eltanamly et al., 2021). Other studies have also shown that refugee children of parents with histories of traumatization report higher rates of common mental disorders and higher levels of child hyperactivity, and conduct, emotional and peer problems compared to children whose parents did not report significant trauma (Sangalang and Vang, 2017; Bryant et al., 2018; Eruyar et al., 2018). A significant insight provided by the trauma framework is that physiological and psychological responses, which were initially protective, can subsequently become hypersensitive, leading to an impairment of the person’s ability to function in everyday life (Nelson et al., 2014). However, the focus on assessing psychiatric diagnoses and emotional and behavioral issues in the literature and studies on refugee populations has drawn criticism for advocating a narrow understanding of mental health and development, thereby potentially oversimplifying the complex issues faced by refugee families. Critiques argue that this focus risks detaching refugees from the broader political and sociocultural context of their experiences and the personal meaning they attribute to them (Westoby, 2009; Ingamells and Westoby, 2008; Summerfield, 1999; De Haene et al., 2007).

Despite the established link between chronic adversity, caregiver stress, and compromised parenting, research and interventions targeting refugee and asylum-seeking populations have faced criticism for adopting a “deficit model,” attributing suboptimal parenting not to the strains of adversity, but to a perceived lack of essential knowledge and skills (Miller et al., 2023, p. 72). However, a growing number of studies from various European countries suggests that the structures of the asylum system and the process of seeking asylum adversely impact the autonomy and parenting role of asylum-seekers. The uncertainty about the future and prolonged processing times of asylum applications contribute to parental distress, which in turn affects the children within these families (Merry et al., 2017; Vitus, 2011; Lietaert et al., 2020). For example, in a study focusing on Danish asylum centers, they found that parents ‘lose their authority over themselves, their children and family life in general, through their total lack of control over both everyday life and future planning’ (Vitus, 2011, p. 105). Similarly, ‘lack of control over housing and the household economy’ can make parents less able to meet their children’s needs in the way they seem appropriate (Ottosson et al., 2017, p. 433). A study from Denmark also found that that asylum-seeking parents’ agentic capabilities to take care of their children are tightly constrained by their housing constrictions and living conditions, insufficient money allowances, regular relocations and juridical status as asylum-seekers (Barghadouch et al., 2022).

In summary, research findings propose various intergenerational processes and contextual factors that affect the quality of parent–child relationships and children’s psychosocial development (Mooren et al., 2023). However, the impact of the migration trajectory and how the process of reestablishing daily family life can challenge parenting and parent–child relationships is still understudied and undertheorized (Shapiro, 2022; Gewirtz et al., 2022). Studies are needed to highlight the narratives of parents in asylum-seeking families, bringing attention to their historically overlooked issues and daily experiences (Saltsman and Majidi, 2021; Merry et al., 2017). Furthermore, there is a need for studies applying theoretical and methodological approaches that place individuals within their specific contexts and examine their interactions within a broader systemic framework (Lustig, 2010; Mooren et al., 2023). This article is part of a larger mixed method study seeking to identify resilience promoting and resilience inhibiting factors, on individual and contextual levels, among asylum seekers and refugees living in reception centers in Norway and Serbia. From the perspective of parents, we aim to understand how the legacy of their refugee background and their present circumstances in exile affect their well-being and parenting practices. Our aim was to gain insight into how mothers and fathers living in the confined context of reception centers in Norway, experience their situation as parents, to make visible their efforts and the coping behaviors they negotiate shaped by distinct temporal, structural and spatial dimensions (Schraube and Osterkamp, 2013) of the reception centers.

## Materials and methods

The study was guided by the framework of Interpretative Phenomenological Analysis (IPA), a method rooted in phenomenology, hermeneutics, and idiography (Smith et al., 2009). IPA focuses on understanding lived experiences from the participants’ perspectives rather than objective accounts (Flowers et al., 1999) and investigates the participants’ concerns and orientations toward the world through their claimed experiences (Larkin et al., 2006). Hermeneutics, the second foundation of IPA, emphasize the research process as dynamic and interpretative (Smith, 1996). Researchers engage in a double hermeneutic process, making sense of participants making sense of their experiences (Smith et al., 2009). IPA’s idiographic focus aims to provide detailed and nuanced analyses of specific cases. We chose IPA as migration research needs to be situated and contextualized (King, 2018) and always placed within a given process (Zapata-Barrero and Yalaz, 2022). IPA’s phenomenological and idiographic approach enables the exploration of participants’ perspectives and the interconnected relationship between the individual and their world (Larkin et al., 2006, p. 117).

### Theoretical framework of the study

IPA also enables the incorporation of diverse analytic strategies from various theories and research, as long as they remain anchored in phenomenological principles (Larkin et al., 2006). The study is embedded in theoretical perspectives that harmonize well with an understanding that people can only be properly understood as functions of our various involvements in the world. We rely on socio-ecological models that emphasize the importance of placing people’s development in context (Bronfenbrenner, 1979) and that focus on children and parents as they develop in real-world settings. Parenting practices are shaped by complex interactions within and between various systems, including the individual, family, school, neighborhood, community, and broader cultural, historical, and political contexts (Betancourt and Khan, 2008; Pacione et al., 2013). These interconnected systems mutually influence each other, impacting adaptation and functioning at all levels (Motti-Stefanidi et al., 2012). In line with this, this study acknowledges resilience as a multifaceted process that transcend individual capabilities, affected by a complex interplay of factors. Resilience is here defined as “the capacity of a biopsychosocial system (can include an individual person, a family, or a community) to navigate the resources necessary to sustain positive functioning under stress, as well as the capacity of systems to negotiate for resources to be provided in ways that are experienced as meaningful” (Ungar, 2019, p. 2). While individual capabilities are an important part of resilience, they are situated within and influenced by a broader context of interpersonal, environmental, cultural, structural, and psychological factors. The term “navigating” refers to the process of finding one’s way, managing, or dealing with a particular situation or set of circumstances. It involves making choices, decisions, or taking actions to successfully move through or overcome challenges, obstacles, or uncertainties. This approach requires moving beyond a simple view of structure versus individual. Contexts like asylum centers, neighborhoods should not be seen as external factors that merely influence individuals. The characteristics of these contexts, such as power hierarchies, shape human development and functioning and should be understood as integral to individual psychological experiences (Mclean et al., 2024).

In addition, the study builds on a sociocultural framework (Bruner, 1990; Rogoff, 2003) that views resilience and parenting as a socially, culturally and historically situated practices. We define parenting practices as behaviors that parents engage in to influence and support their children’s emotional, social, and cognitive development (Eltanamly et al., 2021). Parenting, while a present ongoing process, is strongly influenced by past personal experiences and future expectations (Haavind, 2001, p. 3). Our point of departure recognizes that parents’ aspirations for their children’s development – ‘what is regarded as mature or desirable – are shaped by cultural traditions and practices, as well as the resources and opportunities available in their communities (Rogoff, 2003, p.18), described by Hundeide (2005) as the sociocultural trajectories available for children in a specific time and place. Rogoff (2003) introduced the concept *guided participation* to illustrate how parents serve as facilitators, aiding their children in comprehending and navigating the norms and expectations and requirements of their sociocultural community. However, the material conditions of a family also play a significant role in shaping parenting practices. Economic, social, and environmental factors influence the choices and opportunities available to parents, which in turn affect their ability to protect, guide and support their children’s development.

The study is anchored in the understanding that resilience and parenting cannot be measured by using timeless and context-free standards (Haavind, 2001). Instead, it necessitates exploration through the lens of what Haavind (2011) describes as ‘*what persons are up against and what they are trying to accomplish*’ and to ‘*the contradictions and ambiguities in which persons proceed with in their lives*’ (Haavind, 2011, p. 1). Furthermore, this study does not aim to isolate ‘variables’ and ‘factors,’ but rather seeks to develop knowledge about common issues and social living conditions (Kousho, 2016), while also recognizing the participant’s diversity and individuality (Greene and Hogan, 2005). Living in reception centers is a stressful and uncertain situation, and resilience involves agency and one’s capacity to reflect on everyday conditions and navigate to change one’s circumstances (Triandafyllidou, 2019).

### The research context

This study is set within the context of reception centers in Norway. Upon arrival in Norway, asylum seekers submit their applications and then face a waiting period for the decision, which can span from months to years. This duration varies based on factors like the number of arrivals and the asylum seekers’ country of origin. The organization and management of reception centers in Norway involve collaboration between government authorities, municipalities, and various organizations. Government agencies, such as the Norwegian Directorate of Immigration (UDI), oversee overall coordination, while municipalities play a key role in providing support and long-term integration services. Reception centers are in all parts of the country, and while the activities offered may differ, it is common for Norwegian language courses to be provided. Limited hours of daycare services are generally available for children. Asylum seekers are members of the National Insurance Scheme and are therefore entitled to the same health services as nationals (Helsedirektoratet, 2017). Most of the reception centers in Norway are equipped with the presence of local nurses or health teams, and primary health care functions (Helsedirektoratet, 2017; Rambøl, 2016). Nevertheless, there are significant obstacles that exist, both formally and informally, when it comes to accessing healthcare, particularly mental health services (Varvin and Aasland, 2009).

### Recruitment and participants

The study is part of a larger mixed-method research project with participants (n178) recruited in Norway and Serbia focusing on investigating resilience-promoting and resilience-inhibiting factors on individual, familial, and contextual levels for asylum seekers during their stay in respective countries. Within this broader study, we conducted a separate sub-study concentrating on parents in Norway which is the focus of this article. This targeted approach allowed us to write a separate article based on the analysis of these interviews, providing a detailed examination of the specific issues relevant to parents in these settings.

To provide information about the study, information meetings were conducted in all the reception centers, with translators available to facilitate communication. Individuals who wanted to participate in the study, either volunteered themselves or were recruited through the center administration, staff members, or through casual conversations during the researchers’ visits to the reception centers. The inclusion criteria for the larger study were being a refugee or asylum seekers above 18 years of age living in an asylum reception center. Individuals below 18 years of age or those who were considered too ill to participate in interviews were excluded from the study. We also sought variation in age, gender, family situation, education level, and ethnic affiliation. This approach ensured inclusion of families with children, which is the primary focus of this article. For this sub-study we specifically selected interviews with 12 parents. The parents included came from countries in the Middle East and East/Central Africa. All participants perceived themselves as refugees, and all had applied for asylum. Length of stay in Norway varied (3 months to 10 years). There are both two-parent and single-parent families as well as both mothers and fathers included in this study, and we have interviewed one parent in each family.

### Data production

The data production lasted from 2016 to 2017, altogether 18 months. The Individual interviews took place in a quiet room at the asylum reception centers and lasted 1–3 h. All interviews were audio-recorded with participants’ consent and transcribed verbatim. The researchers conducting the interviews were trained health personnel with experience working with individuals from refugee backgrounds. We carefully adjusted the interview process according to participants’ body language and visible signs of distress, offering breaks and support when needed. Additionally, a team of trained psychologists was available to provide support if necessary. Translators were engaged either through phone or in person during interviews, utilizing interpreters familiar from one of the researchers’ clinical work with refugees. This familiarity ensured that the translators were knowledgeable about our research aims and objectives and the desired interview conduct. Over time, the interpreters also became acquainted with the entire research group, contributing to a more cohesive and trusting environment during the interviews. If the participants were comfortable with English or Norwegian, participants could choose whether they wanted an interpreter, allowing them to express themselves in the language and context (with or without interpreter) they felt most comfortable with, even if they were not fluent in Norwegian or English. Occasionally, participants used both languages during the interview, and we managed this by asking for examples, clarifications, by paraphrasing questions and sometimes mirroring.

To ensure the collection of comprehensive and contextually rich data, multiple sources were utilized. These included audio recordings of interviews, field notes detailing observations and interactions, as well as reflexive accounts to provide in-depth insights into the study. Using this diverse range of data sources allowed for a thorough and comprehensive analysis of the research findings. The researchers also spent time with the residents in the reception centers by participating in daily activities which also facilitated informal and formal conversations/interviews with the families. Field notes gave information into the interview setting, participants’ reactions during the interview, and researchers’ reflections following the interview. This gave opportunity to move beyond selective perceptions and discover issues that were overlooked during interviews. This contextual knowledge also facilitated a better understanding of what had been expressed during the interviews (Fangen, 2010).

### Interview guide

A short, open, semi structured interview guide was developed. The guide focused on resilience inhibiting and resilience promoting processes *prior* to their flight, *during* the flight, and *after* their arrival, for example; *Can you tell me about the period after you arrived in Norway. Can you tell me about a difficult experience? Can you tell me about a good experience?* The interview guide encouraged the participants to share their personal narratives encompassing challenging and supportive experiences. We sought to generate rich, nuanced material from detailed explorations of individual lives (Smith et al., 2009). At times, it was necessary to abandon the structure of the interview guide and follow the participants’ concerns (Smith et al., 2009). Participants were also asked to provide examples of situations (both good and bad), persons, or activities that have had an impact on them during their refugee journey and after arriving in Norway. Viewing the interviewees as participants influenced the interview arrangement. This includes trying to reduce the hierarchy between researcher and participant by providing information and choice and creating an emotionally compassionate and supportive setting. The goal was to create a respectful, empowering environment where they felt in control of their stories.

### Ethics

Ethical approval for the study was obtained from the Regional Committee for Medical Research Ethics (REK) in Norway (REK 2016/65). Prior to participation, all participants were provided with information about the study and were asked to provide informed written consent. They were assured that their personal information, including their names and any other identifying details, would be kept confidential throughout the research process. Participants were informed that the data would be presented in a manner that ensured that their anonymity would be maintained. Additionally, they were assured that the research was separate from the asylum application process, and that they could withdraw from the study at any stage without any consequences. To further guarantee confidentiality, fictitious names were used, and any potential identifying information was altered or removed to safeguard the privacy and anonymity of the participants. As researchers we have also tried to be mindful of the terms we use when writing about people and their experiences, and of the inherent risk of reproducing categorizations and assumptions about the participants (Chase et al., 2020). For example, using the term ‘refugee’ acknowledges the (difficult) situation of the population, but it can also be disempowering and lead to universalizing and homogenizing of distinct experiences (Ingamells and Westoby, 2008; Marlowe, 2010; Edge et al., 2014). Such imposed vulnerability can reduce refugees to helpless victims and passive individuals, often neglecting their agency and sense of subjectivity. Although the terms ‘refugee’ and ‘asylum seeker’ are meant to provide protection and support under the Refugee Convention, they have increasingly become politicized labels of exclusion (Freedman, 2019; Ingamells and Westoby, 2008). Thus, this study is informed by perspectives that emphasize parents as active social agents, rather than just vulnerable refugees or victims, as they navigate daily social contexts and engage in meaning-making processes (Thorne, 2007).

### Analysis

The transcribed material was analyzed in line with the theoretical perspectives discussed above and inspired by Interpretative Phenomenological Analysis (IPA) and the six-step analytic process proposed by Smith et al. (2009). IPA provides both theoretical and practical guidelines for the analysis of the extracted data. The method is concerned with trying to understand lived experience, and how participants themselves make sense of their experiences. It focuses on the meanings that those experiences hold for the participants and is therefore suitable for studies concerned with giving voice to a particular group (Smith et al., 2009). The interviews captured a specific moment within the ongoing journey of the families, serving also as an opportunity for participants to reflect on their past experiences and interpret their current life situation within the reception centers. The objective was to gain a thorough understanding of the myriad factors and processes shaping parenthood by immersing in each individual’s unique narrative.

We read and reread all the interviews with the following analytical question in mind:’ *How, and with what resources, do they cope and create a sense safety, meaning and purpose as parents under these circumstances?* The interview transcripts were first analyzed ‘vertically’, focusing on one interview at a time, and then analyzed ‘horizontally,’ generating themes across cases. The themes were further refined and developed through collaborative discussions among the authors. The first author conducted the initial stage of analysis. Following this, the second and third authors independently reviewed the transcripts. All authors then convened to discuss both individual cases and potential connections across cases. IPA recognizes that interpretations depend on participants’ ability to articulate their thoughts and experiences and the researcher’s capacity to reflect and analyze (Baillie et al., 2000). Self-reflexivity allows researchers to acknowledge their roles in shaping the research problem and findings, emphasizing the need for conscious awareness of these influences on the research process. The authors engaged in regular discussions about their personal biases, assumptions, and emotional reactions to identify how these elements might impact the research process and interpretations. This practice is essential for IPA researchers, who are involved in a double hermeneutic process, “trying to make sense of the participant trying to make sense of what is happening to them” (Smith et al., 2009, p. 3). Our objective was to obtain a comprehensive understanding, not representativeness, and in writing the findings we decided on purposive sampling extracts of rich narratives to be able to give an in-depth understanding. In presenting the themes, we aimed to demonstrate how the parental experiences and stories both diverge and intersect.

## Findings

Responses to displacement stressors vary among individuals. For this study, we aimed to explore how parents in reception centers experienced their circumstances. A pervasive theme across all narratives was the profound disruption caused by uprooting and liminality experienced by these families. The parents describe being trapped in a state of uncertainty and chaos due to a lack of stability, structure, and daily routines, as well as uncertainty about the future. These significant life changes negatively impact family relationships, quality of life, and overall well-being. Their mental and physical health, family roles and relationships, and enthusiasm for life are all affected. Parents longed to return to familiar routines and a sense of “normality,” but the asylum system’s procedures obstructed this need.

### Enforced passivity and lack of agency: *“I am losing control for providing a good life for my family”*

While their circumstances had placed them in a position of need, all parents highlighted their abilities and expressed the need to be recognized as part of the community. They also emphasized their right to be involved in decision-making processes regarding their families. The parents described being constrained or trapped by forces beyond their control, while at the same time under pressure to keep a good lookout on life to protect and support their children and family. These circumstances resulted in feelings of powerlessness – not only when major violations occurred, but in everyday interactions. The erosion of personal agency limited their capacity to care for and support their children. Feeling responsible but without authority to make decisions was described as an unresolved position which was leading to extreme frustration, and the parents were coping with it in different ways. In this sample two fathers described a kind of resistance, a constant desire to gain a greater control by seeking to navigate options available. They were investing considerable physical, mental, and emotional energy into trying to change and deal with their situation. One of them had become a spokesperson for his children and family, but also for other residents. He shared a situation where he went to the staff to try to help a mother get a pram for her child:

I said, she needs a pram, she needs to get a pram. Then they said to me that I do not have respect, and that I complain. So, then I went to the boss, and she told me I have behavioral issues. She used to threaten me with writing a report to the immigration authorities and send us back. My children will never forget those names (Jamal).

What was especially upsetting to him was the loss of autonomy and the way they were moved around from center to center, making it hard for the children to make friends. He felt deprived of the possibilities to make decisions that would support the development of his children and his wife. He took time with describing his experiences:

There is no stability. We move from place to place. We came here to give our children a better life, but there is no stability. There was war for 5 years, so the children never learnt anything. And here we have moved and moved, and they have not learnt anything in Arabic or Norwegian. And they talk about Children’s rights and Human rights (Jamal).

Another father described a similar situation. Seeing himself as the provider and the protector of his family, he was trying to keep his family located close to his brother who was living in Norway, and which was a great support for the children. However, this led to threats from the staff about contacting the child protection agency:

Finally, they threatened me with this organization which takes children. They threatened to take my children, so I came here involuntary. No one listens to us. No one listens to us. We want to share how we feel, but no one listen to us (Omar).

The feeling of having no voice and experience of being treated like a disobedient child or criminal when attempting to take care of their families was emotionally difficult. They were struggling to maintain a position as a parent with authority to make decisions they believed were in the best interest of their child, while dealing with a sense of being degraded. Whilst they described “managing” or “coping” with the situation, they also described the situation as emotionally exhausting. Omar experienced intense pressure from the moment he arrived in Norway, a strain that he found even more challenging than the flight itself:

To be honest, the situation in Norway, it has more pressure than all our previous journeys. My journey, ok it was bad, but compared to our situation now, with the pressure I have been facing the last couple of months, it is nothing. […] we start to get furious and frustrated a lot. Even sometimes I am not talking, I am shouting. I am trying to keep together, not to collapse. But, once you feel angry, you do not know what you are doing. You will lose control. And this is another thing I hate. I hate to lose control. I feel that somebody else is taking the lead of my life and I cannot reach them (Omar).

Whilst some of the fathers where vocally presenting their needs and concerns to make their situation better, other parents resorted to other strategies like keeping silent or entrusting in religion, or trying to keep a positive appearance to protect spouse and children:

I have started to lie a lot, just to hide. I am feeling ashamed that I am lying to my life partner. She is my wife. […] my wife yesterday she was crying, and I am just you know hugging her and telling her I will always find solutions. But I do not have one. But I am trying as usual. I feel like I am losing control (David).

Other parents were also describing this struggle to keep positive in the face of extreme hardship. Esther was a single mother, and she described how her child was totally dependent on her, as it only was the two of them. This led to an immense pressure on her to cope with the situation:

Sometimes I ask myself how I manage, because it is very tough and hard. Personally, I get so many emotions, frustration. But I must be strong, not to show my daughter that I am weak, because she depends on me (Esther).

All parents described that the situation was beyond their control and that they were unable to resolve the situation; they had reached a stage of *impasse; a situation in which no progress seems possible.* This prompted strong feelings of powerlessness and helplessness. Many listed different psychological and physical symptoms, which they believed were mental and bodily responses to their distress. Despite significant adversity, most parents conveyed their ability to “carry on with life.” However, a few seemed on the verge of giving up. A single mother described her situation as unbearable:

I came here and now I hate myself and I do not think I will survive if this keeps up. I will not (Claire).

She detailed the effects of her circumstances, which had led to enduring headaches, difficulties with eating, and a profound sense of restlessness. She also felt an overwhelming urge to flee from certain situations. Her account provided an insight into the profound psychological and emotional toll that the circumstances had taken on her overall well-being. Bahar, another single mother, explained how the constant threat of being deported had resulted in episodes of sudden fear that would impact her children:

Last week I started having these problems. I get scared when I am out with my children. I can start talking loudly, and if I see a truck, I can suddenly get scared (Bahar).

Many of the parents kept these feelings from their families and professionals, which made them feel intensely isolated. They explained how they were trying to remain strong, and to constantly put on a brave face and ignore or even hide their own emotional struggles from family and others.

### Lack of social support and connections: *“Here you are on your own as a mother, no sister, no brother, no neighbors that help you”*

The parents describe a situation where the challenges and stressors they face outweigh the available resources and support systems. The demands and responsibilities exceed their emotional, mental, and physical capacity to cope effectively. While many emphasized their struggles with economy and housing and how these challenges significantly influence the situation of the family, what seemed to generate significant stress and impact health, well-being, and overall quality of life was the feeling of being isolated and unsupported. They long for their previous lives and the social connections they had, while at the same time finding it difficult to form new relationships. They described lack of networks that could provide emotional, informational, and tangible support, and help them cope with stressor of everyday life. Many talked about how they in their home countries used to be able to draw on immediate and extended family as well as neighbors and community for help and support in caring for their children. The absence of such extended family ties resulted in feelings of uncertainty and isolation. Some felt overwhelmed by the responsibility of looking after their children without the support of intergenerational and communal care systems that they had access to back home. As Jamal expressed it;

In my homeland I had the support of my family, both grandmothers, and the rest of the family. Aunts and uncles, they were all in the same area. I did not worry about anything with the children, either education or upbringing, because we were surrounded by people, and I could concentrate on work. But here, everything is on me. Everything is on me now (Jamal).

Jamal reflects on the significant differences between his life in his homeland and his current situation. Back home, he had the support of a close-knit community that provided a sense of security and shared responsibility. In contrast, his current situation places the entire burden of responsibility on him, as emphasized by his repetition of “everything is on me now.” This underscores his feelings of isolation and the overwhelming pressure he faces without the familial support structure he once had. Lack of network was also reflected in the interview with Bahar;

I am worried about my children. Who can take care of them. I am a single mother. I have no one (Bahar).

This quote poignantly captures her deep sense of anxiety and isolation and reflects the immense responsibility she feels as their sole caregiver. While one of the parents described how they received some support from another family living in the reception center, the overarching theme in the parent narratives was the feeling of being on your own:

It is a big difference. A big difference. You know in my home country I was quite young when I had my son, but my mother was there for us and in many ways, she raised my son. So, you have a lot of support from family back home, but here you are all alone. No sister, no brother, no neighbors (Esther).

The narratives revealed a profound sense of isolation and detachment. It was not merely about being physically alone but described as a feeling of being emotionally disconnected. They expressed a deep longing for supportive, reciprocal relationships with other adults and trustworthy friends. However, pervasive insecurities about the intentions of others often obstructed the formation and maintenance of meaningful connections. This underlying distrust severely limited their opportunities for genuine social engagement and interaction. Esther explained;

Interviewer: Okay, is there anyone here you trust? Do you have any friends?

Parent: I do, but friends are always friends. You never trust them.

Interviewer: So, you do not trust them?

Parent: They are there, but you can find that they are trying to push you away. They think you are a burden to them. So, if you feel that someone is pushing away, you must backout yourself.

Interviewer: You feel like you are a burden?

Parent: Ya.

The stress parents endure from cumulative pre-migration trauma and post-flight stressors is intensified by a lack of sensitive support and compassion. The parents describe feeling disconnected and isolated as they try to manage their responsibilities, and the complex environment of the asylum center. This combination of pressures can severely undermine mental health, well-being and parenting capacity, making it difficult to maintain hope in an already uncertain situation.

For individuals already experiencing mental health issues, the lack of understanding or validation from others contributed to feelings of self-doubt, as one single mother explained;

You might get friends, but they are not there for you and once you come here at the asylum center people look at you like you are an awful person, I think (Hana).

Hana expresses skepticism about the sincerity of friendships in the asylum center, feeling that these connections lack genuine support. Her experience also give insight into the potential harmful effects of traumatization and stigmatization on self-worth and sense of belonging.

### Being a parent: *it is hard. It is not easy. But becoming a mother, what can I say. It is something that is good*

The children represented hope and the possibility of a better future and for many of the parents, their children were a powerful reason to strive for stability, despite the adversity they encounter. However, they presented being a parent under these circumstances as a juxtaposition between something fundamentally good, and at the same time a source of great concern and distress. The joy of parenthood was often juxtaposed with constant worry about the safety, health, happiness, and overall well-being of their children. All parents describe ensuring a good life for their children as their most important task in life. The children were also described as a rationale to keep positive, and that caring for the children brought a sense of purpose and meaning to their parents’ lives amidst the struggles. Amira, a single mother of two children described becoming a mother as a deeply enjoyable experience, however it was shadowed by a constant fear of being separated from her children:

I have tried to get a Norwegian passport, but now I sit at home doing nothing. Thinking a lot. I get depressed by it. But I am so lucky to have children. Before I had my first child, I used to take sleeping medication. I feel better now because I have my children. And thank God they live in Norway (Amira).

When asked to describe her experience as a mother living in the reception center, she provided a balanced response. She acknowledged that it is challenging, but emphasized the positive aspects, suggesting that motherhood helps her maintain faith in the good things in life:

It is hard. It is not easy. But becoming a mother, what can I say. It is something that is good. If I think about my children, I get happy.

Some individuals also viewed parenthood as a meaningful way to stay occupied and to pass the time while they were in limbo, awaiting a decision on their case. They found that the responsibilities and joys of raising children provided a sense of purpose and helped them cope with the uncertainty and stress of their situation. Being a parent allowed them to focus on something positive and constructive, giving them a reason to stay hopeful and engaged despite the prolonged wait and ambiguity of their circumstances:

Very good to be a dad. I take care of him and that is entertaining. It is very nice to play with him, it is a way of passing time. Children makes you happy (Hassan).

When discussing the possibilities of returning to their homeland Hassan explained how the important thing was to ensure that his child could have a good life and that what happened to him was less important. Caring for their children gives the parents a clear purpose and direction. The desire to provide a better life for their children can be a powerful motivator;

He is a child, so I will stay here and give him a happy life. I can have my miserable life, but he should have a happy life (Hassan).

The inherent goodness and joy of having children coexist with the concerns, challenges, and distress that come with such a significant responsibility. The obligation to ensure a safe, healthy, and nurturing environment for the children when feeling confined in the asylum system, can be overwhelming;

Thank God for my daughter. She is still happy and playing and jumping. And everybody like her. Everybody says that she is always clean, you know, dressing well. But I am not satisfied about that. I know I can give her more than that. But I do not have the power (David).

Many parents were worried about how their situation was hindering the natural development of their children. They feared that their child might not be achieving the expected milestones or making progress in physical, cognitive, emotional, or social development in comparison to their peers. This concern could arise from the child not gaining the skills, abilities, or knowledge deemed age-appropriate for their stage of development, or from their inability to learn the Norwegian language. The lack of activities and socializing with other children was also a great concern;

It is okay here, nothing bad is happening with the child, but that a child spends all his time with grown-ups, that is not good. Children should spend time with other children (Hassan).

Hassan explained that this situation made him very sad, and he expressed concern about the opportunities his child was missing out on, which other children were privy to:

My son is now 1 year, and 7 months and he should be in kindergarten. Other children the same age have been to kindergarten 1 year already. So, when I look at my son, I get sad, because he wants to play and participate in activities.

The parents shared deep-seated fears about their children’s futures. One major concern is the uncertainty surrounding their legal status and the possibility of deportation, which can disrupt their children’s sense of stability and security, however the parents also worry about the impact of prolonged stays in asylum centers, where access to quality education is limited, potentially hindering their children’s psychosocial and academic development and future opportunities.

## Discussion

Refugee parents have the challenging task of providing not only the basic needs of their children but also supporting their psychological and social needs in a new and unfamiliar, and what also often can be described as family hostile environments. This study focuses on the experiences of parents in asylum centers in Norway. The explorative design of the study, and the focus on resilience inhibiting and resilience promoting processes generated insights into common issues and social living conditions (Kousho, 2016) experienced by the parents. This study recognizes the agency of refugees, understanding that they actively engage with and respond to their environment. Our study provides valuable insight into how external factors, such as restrictive processes and structures, influence parenting practices among refugee parents. It also give insight into the varied responses and coping styles of the participants and how the parents strive to impact their own outcomes and surrounding conditions. Despite adversity, trauma, and challenges, they view parenthood as a source of hope and purpose.

Understanding the psychosocial aspects of parental well-being is crucial, as it affects not only the parents but also influences child development (Eltanamly et al., 2021). The findings align with previous research showing that forced migration causes significant changes in life trajectories that build up and persist through the stages of preflight, flight, exile, and resettlement. Parents face parenting challenges for which they lack pre-existing strategies (Gillespie et al., 2022). This study examined the context of Norwegian asylum centers, revealing an experience of structural violence (see Galtung, 1969) described by the parents as form of violence that emerged through bureaucratic routines that adhere to policies ignoring individual needs and depriving the parents of basic parental rights, including the right to protect and care for the child’s safety and well-being. This involved creating a safe environment, addressing their emotional and physical needs, and shielding the children from harm or neglect. The parents described in detail how constraints imposed by the asylum system acted as roadblocks, hindering them from tapping into and utilizing vital internal and external resources, mirroring findings in the critical review by Hynie (2018). Understanding refugee protection just as protection from physical violence may hinder the recognition of such harm experienced by refugees’ post-settlement. National resettlement systems are often shaped more by the needs and preferences of the host society than those of refugees (Rafferty et al., 2020; Phillimore, 2021). This limited perspective leads host societies to believe they have met their humanitarian obligations merely by granting legal residency, while ignoring systemic barriers to social and economic participation that can harm mental health similarly to pre-migration physical and mental violence (Hynie, 2018; Porter and Haslam, 2005; Rafferty et al., 2020). Protection should focus on preventing the accumulation of post-flight stressors that impede recovery and the primary goal should be to provide a “safe haven” that facilitates psychosocial rehabilitation (De Haene et al., 2010).

The parents in our study describe an attachment system fragmentation (Liddell et al., 2022) which makes the whole family vulnerable. There was a lack of close relationships that could buffer the effects of stress and provide a sense of security and support, and an important finding is that the lack of social support, led parents to rely on individual coping mechanism. This lack of social support and disruptions to their attachment systems, and high-effort coping, resulted in exhaustion and loss of hope, like experiences highlighted in a study from Canada (Simich et al., 2005). Such experiences of significant social stressors in the postmigration environment—including isolation and loneliness—can impede recovery from experienced traumatization (Liddell et al., 2021), and many of the parents experiences severe mental health stress related symptoms. This, in turn, can further impair their capacity to respond effectively to their children’s emotional needs. It is documented that war exposure and post-migration stressors can increase parenting stress and lead to mental health issues that impact parenting practices (Eltanamly et al., 2021), making it difficult for parents to regulate their own emotions, which in turn affects their ability to regulate their children’s emotions. This can lead to decreased sensitivity, less structure, and increased hostility and harsh parenting (Bryant et al., 2018; van Ee et al., 2012).

Our study provides significant insights into the reciprocal and dynamic relationship between refugee families and their environments, reinforcing the importance of viewing refugees’ experiences as part of an interconnected system where individual and environmental factors are interdependent. The literature highlights that secure attachments are crucial for mental health and serve as key emotion regulation strategies to mitigate the adverse effects of forced migration on family life (De Haene et al., 2010). Emphasis should therefore be on creating supportive social conditions for healthy parent–child relationships, and host societies should aim to heal traumatic responses to restore parental caregiving capacities (De Haene et al., 2010). Both trauma and resilience research agree on the importance of strong emotional attachments for resilience and dealing with or preventing the negative consequences of being traumatized. Our study give insight into a double negative; while the structures of the asylum system hinder social inclusion, in line with Baumgartner et al. (2024), our study also give insight into how traumatization may make individuals less likely to engage, further hindering their participation.

A growing amount of research find long-term impact of war exposure on parenting practices and child adjustment, however there is still a need for research that aim to address the mechanisms through which war exposure affects parenting practices (Eltanamly et al., 2021) and that consider the whole process of preflight, flight and postmigration phases. This study shows that the capacity of parents to support their children depends on a complex interplay of processes on multiple levels, and that attention in research and practice needs to incorporate a socioecological perspective (Bronfenbrenner, 1979; Miller and Rasmussen, 2017) when assessing parenting practices and parent–child relationships. This requires attention to the broader psychosocial and socio-political and cultural contexts, as well as the persistent pre and post-migration stress that families endure, including traumatization, marginalization, socioeconomic disadvantage, acculturation difficulties, loss of social support, and cultural bereavement (Wachter and Gulbas, 2018; Reed et al., 2012; Miller and Rasmussen, 2010; Eisenbruch, 1991; Silove et al., 2017; Summerfield, 1999; Marlowe, 2010). The focus on resilience in this project allowed an exploration of how social arrangements and relationships—which provide people with inner security, a sense of stability, human dignity, and a sense of meaning and purpose—are affected by war, forced migration and the structures of the asylum system (Sideris, 2003; Silove, 2013). The study supports the understanding that sub-optimal parenting among refugee parents is not primarily due to a deficit in knowledge and skills, but rather to a sense of disempowerment, helplessness, and persistently heightened levels of stress adversely affecting their parenting capacity (Miller et al., 2023; Eltanamly et al., 2021). Resilience involves actively and thoughtfully maneuvering through situations, assessing circumstances, understanding available options, and making informed decisions (Ungar, 2019). However, the lack of social support, enforced passivity, and dependence on others hinder the capacity for meaningful adoption of supportive strategies (Rizzi et al., 2023; Menéndez Álvarez et al., 2021; Sagbakken et al., 2022). In line with other studies focusing the situation of parents in asylum centers in the welfare states of the Nordic countries (e.g., Vitus, 2011), this study shows how the asylum system challenges parental opportunities to care for their children.

The study is based on a thorough and comprehensive methodology that incorporates multiple data sources, including interviews, field notes, and reflexive accounts, providing a rich and nuanced understanding of the experiences of the families involved. The use of semi-structured interviews allowed for flexibility and openness, enabling participants to share their personal narratives and experiences in their own words. While this study offers valuable insights into the factors that promote and inhibit resilience among asylum seekers in reception centers, several limitations must be acknowledged. The inclusion of a diverse participant group, encompassing variations in age, gender, family situation, education level, and ethnic affiliation, may enhance the transferability of the findings. However, this lack of homogeneity can also present a significant limitation. The study includes both two-parent and single-parent families, which may have different dynamics and challenges that affect their resilience and well-being. Additionally, the participants’ length of stay in reception centers varied significantly, ranging from 3 months to 10 years. This variation could impact their experiences and perceptions, as those who have been in the reception centers longer may have different coping mechanisms compared to those who have recently arrived. Age and ethnicity are other factors that were not homogenous among the participants. The study included parents from diverse age groups and ethnic backgrounds, specifically from countries in the Middle East and East/Central Africa. These differences could influence the resilience and risk factors experienced by the families, as cultural and age-related differences may play a role in their adaptation and well-being. The sample size can also be considered as small and the use of translators, although necessary, could potentially influence the data that were gathered, as nuances of language and meaning could be lost or altered during translation. Their presence ensured that language did not become a barrier to participate but it is also important to acknowledge that the translation process itself might have influenced the data. Translators not only interpreted the spoken words but also conveyed cultural nuances and context, which are integral to qualitative research. As such, the translators’ interrelations with the participants and researchers could have impacted the way information was shared and understood. Conducting research with forced migrants, who represent a culturally diverse group, presents considerable challenges for researchers. These challenges may stem from cultural and linguistic disparities as described above, however when working with forced migrants we as researcher also need to be mindful of how the potential impact of trauma on both internal and interpersonal dynamics may influence the interview process and narratives (Tessitore and Margherita, 2024). During the data collection process, we consistently adopted a reflexive approach, regularly examining how our positions and interactions with participants might influence the research. This reflexivity enabled us to remain cognizant of potential power dynamics and actively work to mitigate them.

We believe the analysis in this study offers a comprehensive and nuanced understanding of the complex realities faced by asylum seeking parents in Norway. We have tried to situate the findings within the broader literature and demonstrate alignment with previous research. Our study underscores that parental resilience is a complex, dynamic process influenced by time- and context-specific factors. Consequently, interventions aimed at fostering resilience must be highly tailored to individual contexts, rather than relying on a universal model that assumes uniform effects across different settings (Tol et al., 2013). The theoretical framework of the study made visible how the hierarchies of power and privilege was shaping parent practices and functioning. Approaches in research that do not acknowledge systemic inequalities and make visible hierarchies of power and privilege by not exploring them can limit our understanding of the experiences of refugee parents and can risk perpetuate “victim-blaming.” Furthermore, interventions aimed at supporting refugee parents and their families need to consider the complex interplay of individual, interpersonal, and systemic factors that impact their experiences and resilience (Hynie, 2018). These interventions should move beyond perspectives that tends to isolate the individual, and family, from the social and political context (Fennig and Denov, 2019; Shapiro, 2022). Further research is needed to explore how refugee parents’ experiences impact the relationship with their children, and child development. Future designs could benefit from incorporating longitudinal data and diverse perspectives, such as those of different cultural and linguistic groups, children, other family members, and community members, to provide a more holistic understanding of the experiences and challenges faced by refugee families. This could involve examining the asylum process, the role of institutional discrimination, and the impact of the socio-political context on parenting practices and well-being. The role of social support in facilitating resilience and well-being among refugee parents should also be explored further, including developing interventions that focusing on providing reciprocal social networks and community support in the resettlement process such as:

- peer support groups where parents can share experiences, offer mutual support, and build a sense of community.
- mentorship programs that connect parents with mentors or advisors who can guide them through the asylum process and offer emotional support.

## Data Availability

The raw data supporting the conclusions of this article will be made available by the authors, without undue reservation.
